# Hospital Contracts and Prices

**Published:** 1910-06-04

**Authors:** 


### Special Departmental Article.

**Contributions and questions on matters affecting this department of an institution are invited.**

### HOSPITAL CONTRACTS AND PRICES.

### VIII.—BEDDING, LINEN, ETC.

There are no articles of hospital consumption which require to be purchased with greater care than bedding, linen, and cotton goods. In the case of bedding, the different qualities of hair for mattresses, and of feathers for pillows, are so numerous that expert knowledge is necessary to distinguish the relative values. The resiliency of the hair used makes a great difference to the life of the mattress and the comfort of the patient, and it is not wise to pay too low a price. If very cheap hair is used a mattress becomes hard and matted, and it is necessary to re-make after a very short time. The loss in weight through re-teasing cheap hair being considerable, the few shillings saved in the original purchase are soon lost and additional expense incurred by the expenditure upon re-making, and after all the mattress remains unsatisfactory. A hair at about 1s. or 1s. 1d. per lb. is recommended, and ticking of good quality. Hospital mattresses have to be subjected to frequent stoving and disinfection, and an inferior ticking will not stand the excessive heat. Care should be taken also to specify that, instead of the usual leather tabs, strong tufts of worsted be substituted, as the leather tabs shrivel during the process of disinfection and get so hot that the ticking is liable to become scorched and rotten immediately underneath the leather. The worsted stands disinfection well and does no damage to the ticking.

The following specification for a hospital mattress will ensure a good article being obtained, it being understood that a ticking of good quality has previously been selected:—

> Mattresses for bedsteads 3 feet by 6 feet 6 inches, made with 4-inch borders piped with ticking and to contain 27 lb. of hair at say 1s. or 1s. 1d. per lb., the ticking to be as sample, the tabs to be strong tufts of worsted.

The price of a mattress made to this specification should be from 38s. to 41s., and a hospital committee prepared to pay this price will not regret the expenditure.

Feather pillows should be specified to have a best quality linen tick to match the mattress in pattern and to contain 2½ lb. of feathers. The price can range from 3s. to 6s. 6d. or 7s., according to the quality of the feathers, but a very good pillow to the specification suggested can be purchased wholesale at from 4s. 2d. to 4s. 6d.

The question of the best quality of blankets for use in hospitals is a vexed one. The prices paid vary considerably. We have no doubt that the various hospital authorities have good reasons for adopting the blanket they have individually selected. One thing that should always be borne in mind is the constant washing to which hospital blankets are subjected, and before deciding to adopt any particular sample it would be well to submit it to a washing test, so as to ascertain the loss of weight and the shrinkage caused by the first washing. The following specification has produced a good serviceable article:—

> White cloth blanket 10/4 by 8/4, weight 3½ lb. single, whipped both ends, and marked (initials of hospital).

The stretched measurements of such a blanket before and after being washed should be as follows:—

> Before being washed, 92 inches by 82 inches.
> After being washed, 90 inches by 82 inches.
> The loss in weight should not exceed 5 oz.

It should be noted that for the purpose of the test it is necessary to stretch the blanket to its utmost capacity both before and after it is washed. The washing should, of course, be carried out carefully by an experienced laundry-hand. The cost varies with the price of wool, but blankets to the above specification should be purchased in quantities for from 6s. 6d. to 7s. 6d. each, with the addition of 1d. per letter for marking.

There is considerable difference of opinion amongst hospital administrators as to whether sheets, pillow-slips, etc., for ward use should be made of linen or cotton, but whichever is determined upon a quality which will stand hard wear is a necessity. Most hospital authorities prefer, if possible, to purchase their requirements locally. Various methods of obtaining supplies are adopted, but an annual purchase of the estimated requirements for the year is an excellent plan if storage accommodation is available. Competition is usually keen, especially in the case of provincial hospitals, local drapers being glad to tender. There is, however, a tendency in these days of cut prices for something just a trifle worse than the hospital's approved patterns to be tendered for. There are so many qualities so nearly alike and so many different manufacturers that it is difficult for the firms tendering to quote exactly to sample unless the manufacturers and the manufacturers' numbers are known, and consequently the nearest to the samples have necessarily to be quoted for, and, naturally, in the face of keen competition, firms cannot afford to quote for slightly better articles and thus diminish their chance of success.

The consideration of the tenders and samples is a difficult task, and a secretary and matron who are good judges of linen and cotton goods are of great assistance to the Contracts Committee, and the presence of an expert upon the committee itself is invaluable.

Much trouble is saved if, when preparing the tender form, the name of the manufacturer and the manufacturer's number or letter describing the quality is inserted after each article. The tendering firms can then quote to the exact requirement, the committee is saved the trouble of examining samples, and the lowest tender can be accepted in perfect safety.

It is a matter for consideration whether it is cheaper to buy materials and make sheets, pillow-slips, towels, etc., in the institution or to buy them ready made. After fully considering both methods we are inclined to the latter. Manufacturers have facilities for making which hospitals do not possess, and, combined with the small saving in cost, there is a considerable saving of worry and work in the linen room.

The following prices of linen and cotton goods, etc., delivered ready made within the last twelve months may be of interest to hospitals which make everything in their own linen rooms:—

| | £ | s. | d. |
|---|---|---|---|
| Cloths, dressing | 0 | 0 | 6 |
| Cloths, glass | 0 | 0 | 7 |
| Cloths, lavatory | 0 | 0 | 4¾ |
| Cloths, locker | 0 | 0 | 6 |
| Cloths, medicine | 0 | 0 | 3½ |
| Cloths, table, kitchen | 0 | 3 | 6 |
| Cloths, table, nurses' | 0 | 2 | 6 |
| Cloths, table, ward | 0 | 4 | 3 |
| Cloths, tea | 0 | 0 | 5¼ |
| Counterpanes | 0 | 8 | 0 |
| Counterpanes, cot | 0 | 3 | 6 |
| Covers, bedpan | 0 | 0 | 8¾ |
| Covers, bottle, hot water | 0 | 0 | 6 |
| Covers, mattress | 0 | 3 | 2 |
| Covers, pillow | 0 | 0 | 7 |
| Covers, screen | 0 | 7 | 1 |
| Covers, tent | 1 | 7 | 6 |
| Dusters | 0 | 0 | 2¾ |
| Gowns, dressing | 0 | 7 | 0 |
| Gowns, operation | 0 | 7 | 0 |
| Jackets, bed, red flannel | 0 | 4 | 3 |
| Nightingales, red flannel | 0 | 3 | 6 |
| Sheets, American cloth | 0 | 1 | 3 |
| Sheets, cotton, bassinette | 0 | 1 | 0 |
| Sheets, cotton, bed | 0 | 3 | 6 |
| Sheets, cotton, cot | 0 | 2 | 4 |
| Sheets, cotton, draw | 0 | 1 | 9 |
| Sheets, linen, mortuary | 0 | 5 | 6 |
| Sheets, mackintosh, bed | 0 | 12 | 9 |
| Sheets, mackintosh, dressing | 0 | 6 | 4½ |
| Slips, pillow | 0 | 0 | 9¼ |
| Towels, bath | 0 | 0 | 11 |
| Towels, hand, doctors' | 0 | 0 | 9¼ |
| Towels, roller | 0 | 1 | 11½ |
| Wringers | 0 | 0 | 6 |

